# Prognostic revalidation of RANO categories for extent of resection in glioblastoma: a reconstruction of individual patient data

**DOI:** 10.1007/s11060-025-04950-0

**Published:** 2025-02-24

**Authors:** Johannes Wach, Martin Vychopen, Erdem Güresir

**Affiliations:** 1 Department of Neurosurgery, University Hospital Leipzig, 04103 Leipzig, Germany; 2 Comprehensive Cancer Center Central Germany, Partner Site Leipzig, 04103 Leipzig, Germany; 3 Department of Neurosurgery, University Hospital Leipzig Leipzig University, Liebigstraße 20, 04103 Leipzig, Germany

**Keywords:** Glioblastoma, RANO classification, Extent of resection, Overall survival, Surgery

## Abstract

**Background:**

The RANO classification for glioblastoma defines resection categories based on volumetric tumor assessments, aiming to standardize outcomes related to extent of resection (EOR). This study revalidates the prognostic impact of RANO classes by reconstructing individual patient data (IPD).

**Methods:**

A systematic review and meta-analysis were performed, including three studies comprising 580 glioblastoma patients. Included studies reported or allowed conversion to RANO classes for glioblastoma resection extent, with detailed OS data and numbers at risk. Overall survival (OS) data were extracted from Kaplan-Meier survival curves, and IPD were reconstructed using Digitizelt and the R package IPDfromKM. Survival analyses were conducted using Kaplan-Meier estimates and Cox regression models.

**Results:**

Median follow-up was 15.6 months (IQR: 10.1–28.8). Patients undergoing supramaximal resection (RANO class 1, *n* = 163) had the highest median OS (35.6 months; 95% CI: 30.9–40.4), significantly outperforming non-class 1 resections (median OS: 13.9 months; 95% CI: 13.0–14.7; *p* < 0.001). Subgroup analysis revealed superior OS for class 2a (19.0 months) over class 2b (14.1 months; *p* < 0.001), while class 3 and 4 resections demonstrated progressively poorer outcomes. Hazard ratios consistently favored class 1 versus all other classes (HR: 0.28; 95% CI: 0.23–0.37).

**Conclusions:**

Supramaximal (class 1) resection provides a significant survival benefit in glioblastoma, underscoring its critical role in surgical management. The RANO classification stratifies resection outcomes effectively, supporting its use as a prognostic tool. These findings advocate for resection strategies targeting maximal tumor removal.

**Graphical Abstract:**

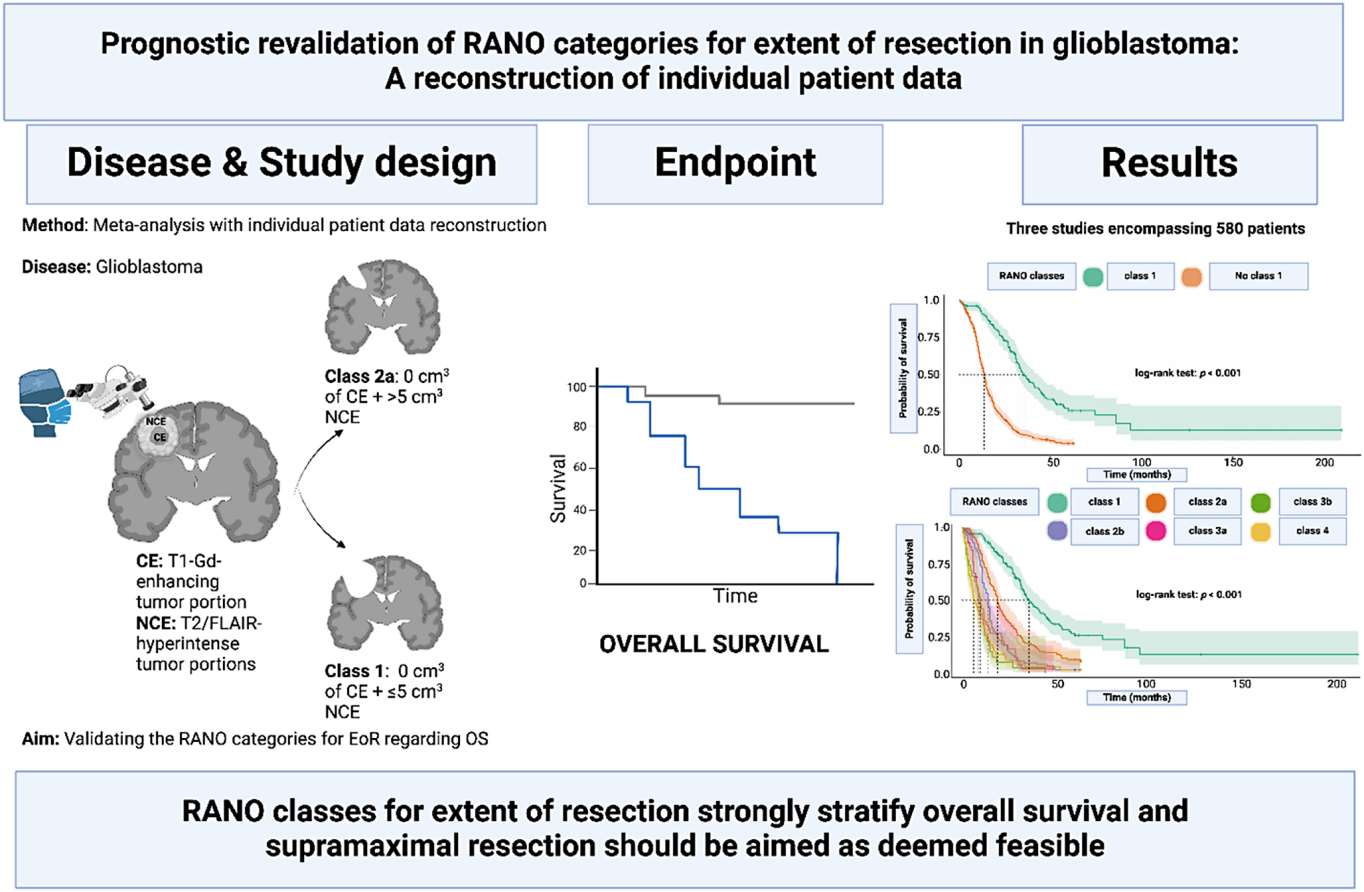

**Supplementary Information:**

The online version contains supplementary material available at 10.1007/s11060-025-04950-0.

## Introduction

Glioblastoma (GB) remains the most common and aggressive primary brain tumor in adults, characterized by poor prognosis and limited therapeutic options despite advancements in molecular profiling and treatment strategies​ [1, 2]. Maximal safe resection is widely regarded as the optimal surgical approach, with different thresholds for extent of resection (EOR) being reported to be consistently linked to improved overall survival (OS) and progression-free survival (PFS) ​ [3, 4]. However, the evolving terminology and classifications, including the RANO (Response Assessment in Neuro-Oncology) resection classes and supramaximal approaches, highlight the need for standardized thresholds to better define survival benefits and surgical outcomes [5].

Emerging evidence highlights the survival benefits of supramaximal resection techniques (e.g. FLAIRectomy, lobectomy, safety margins) and gross-total resections, particularly for IDH-wildtype GBM, aligning with the updated WHO classification​ [6]. The RANO resect classification offers the first standardized framework for categorizing EOR of contrast-enhancing (CE) and non-contrast-enhancing (nCE) tumor portions with supramaximal resection techniques based on residual tumor volumes, facilitating more precise correlations between surgical interventions and oncological outcomes​ [5]. While prior studies have underscored the importance of residual tumor volume as a predictor of survival, variations in methodology and patient heterogeneity necessitate further validation of these classifications​ [3, 4].

Given the critical prognostic implications of EoR in GB and the increasing adoption of novel classification frameworks like the RANO system, revalidation of these powerful models through independent pooled studies is essential to ensure their generalizability for further studies and reliability across diverse patient populations. In this meta-analysis, we aim to revalidate the prognostic utility of RANO resect classes in GB by pooling and reconstructing individual patient data (IPD) from multiple studies. By integrating survival data with RANO classifications, our analysis seeks to provide robust evidence on the impact of EOR on clinical outcomes, thereby refining surgical strategies and informing future clinical trials.

## Methods

### Search strategy and data collection

The present meta-analysis adhered to the PRISMA guidelines (see PRISMA checklist, supplementary (see supplementary methods 1) for systematic reviews and meta-analyses and was prospectively registered in the International Prospective Register of Systematic Reviews (PROSPERO, ID: CRD42024615859) [7]. Individual patient datasets (IPDs) were extracted from PubMed, Google Scholar, and Cochrane library. Literature search started after the publication of the RANO resect classes and was performed between August 12, 2022, and November 1, 2024. The search utilized both MeSH and non-MeSH keywords, including “glioblastoma,” “RANO resect”,”RANO classification”, “Surgery”, “extent of resection,” and “survival”. Details on the search syntax are summarized in supplementary methods 2. Only English-language articles were included. Two independent researchers searched and screened the literature. The detailed study protocol is given in supplementary methods 3.

### Inclusion criteria

Inclusion was limited to English-language studies on humans with detailed OS data and numbers at risk, focusing on patients aged 18 years or older with an initial diagnosis of histopathologically confirmed glioblastoma. Volumetric assessment or radiological of the EOR based on T1-contrast-weighted and FLAIR MR-images should have been performed. These data should be directly described as RANO classes or transferrable to RANO classes.

### RANO resection classification

EoR for stratification of OS was performed based on the RANO resect classification. The RANO classification for glioblastoma resection defines four main categories based on the extent of CE and nCE tumor resection. Class 1 (Supramaximal CE Resection) involves complete removal of CE tumor with ≤ 5 cm³ nCE residual volume. Class 2 (Maximal CE Resection) includes two subcategories: 2 A (complete CE resection, > 5 cm³ nCE) and 2B (near-total CE resection, ≤ 1 cm³ CE residual). Class 3 (Submaximal CE Resection) also has two subtypes: 3 A (subtotal CE, ≤ 5 cm³ CE) and 3B (partial CE, > 5 cm³ CE residual). Class 4 (Biopsy) reflects no significant tumor resection [5].

### Quality assessment

The methodological quality and potential biases of the included studies were assessed using the NIH Quality Assessment Tool for Observational Cohort and Cross-Sectional Studies (NIH-QAT). This tool offered a structured framework for systematically identifying the strengths and weaknesses of each study [8].

### Data extraction & individual patient data reconstruction

Two authors (JW, MV) independently collected data on variables such as age, sex, MGMT promoter methylation, IDH1 status, Karnofsky Performance Status (KPS), preoperative tumor volume, EOR, and the application of adjuvant radiochemotherapy. Discrepancies between the authors were addressed through re-evaluation or by consulting a third author (EG). OS data and the number at risk were extracted from Kaplan-Meier survival curves using Digitizelt software (Version 2.5.10 for macOS). The extracted data were then utilized for individual patient data (IPD) reconstruction with the R package IPDfromKM [9, 10]. Table 1 provides an overview of the included studies and their patient characteristics with matching criteria for extracting data.

### Statistical analysis

IPD from all studies were pooled to construct Kaplan-Meier curves for OS, stratified by each RANO class. These analyses were conducted using the R packages *Survminer* and *Survival* in version 4.3.1 (R Foundation for Statistical Computing, Vienna, Austria). Twelve-, 18-, and 24-month survival rates for were calculated. Subgroup comparisons for OS were analyzed using the log-rank test, applying a significance threshold of *p* < 0.05.

Subgroup analyses comparing each RANO classes with each other using Cox regression analyses reporting unadjusted Hazard ratios were performed. Forest plots illustrating these results were created with the R package *ggplot2*.

## Results

### Search results and included studies

**Fig. 1:**
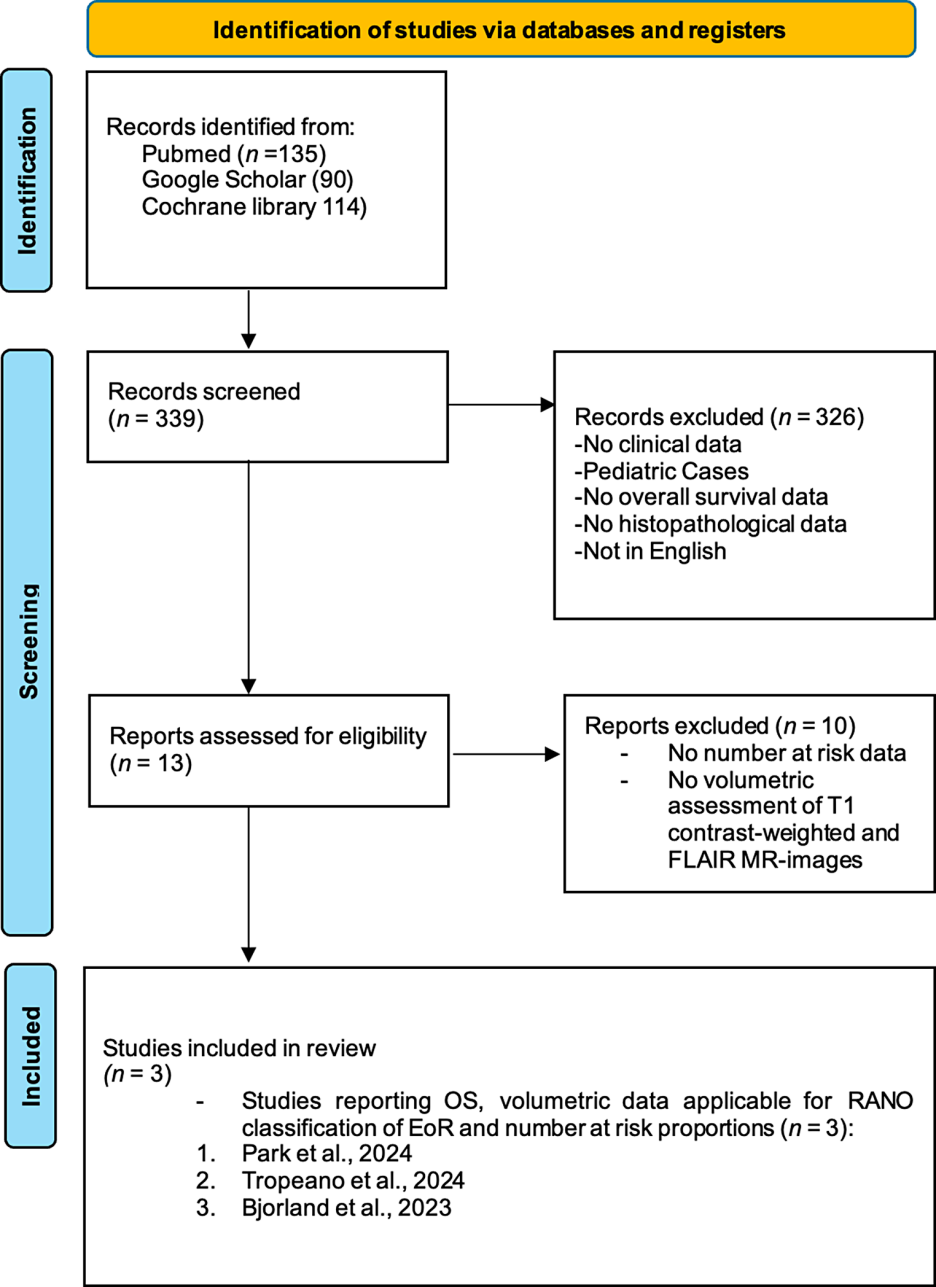
PRISMA flowchart for study selection

The initial search in Pubmed, Google Scholar, and Cochrane library identified 339 studies. Based on the abstracts, 326 studies were excluded. The remaining 13 studies underwent a detailed review. Of these, 10 studies were excluded due to lack of OS data stratified by EoR in line with RANO classes. After a thorough analysis, 3 investigations were included in the meta-analysis (see Fig. 1).

Tropeano et al. [11], a single-center retrospective study from Italy with 117 patients, reported a median age of 63 years, 65.8% MGMT methylation, and high preoperative Karnofsky Performance Status (KPS, mean 91.6). Bjorland et al. [12], based in Norway, analyzed 235 patients in a population-based cohort with a median age of 62.6 and did not report MGMT methylation rates or KPS. IDH1 status was not routinely tested in this study but younger patients and those with histological features of low-grade glioma were investigated regarding IDH1 mutation and those patients were excluded. Tropeano et al. [11] and Bjorland et al. [12] directly applied the RANO resection classification. Park et al. [13], a multicenter study in Korea and Germany with 1,158 patients, found 40.6% MGMT methylation and a median KPS of 90. This study provided a multifactorial classification stratification system based on volumetric EoR, MGMT promotor methylation, age and KPS. All studies used the Stupp protocol as a standard, though variations in chemotherapy and radiotherapy adherence were noted. The study by Park et al. [13] not directly adapted the RANO classification but their Class 1 patients definitely underwent a complete resection of both CE tumor portions and NCE tumor portions, which implies that they are RANO class 1 (0 cm [3] CE + ≤ 5 cm [3] NCE). Class 2 and 3 patients of these study were not included because there could be also RANO class 1 resected patients among these groups. Therefore, 163 IPD of their Class 1 constitute RANO class 1 patients and 106 of their Class 4 patients have not undergone a RANO class 1 resection. Table 1 summarizes the key characteristics of the included studies.

**Table 1:** Characteristics of the included studies

Study Characteristics	Tropeano et al.^11^, 2024	Bjorland et al.^12^, 2023	Park et al.^13^, 2024
Study Design	Retrospective single-center cohort	Retrospective population-based cohort	Multicenter retrospective cohort
Country	Italy	Norway	Korea, Germany
Total number	*N* = 117	*N* = 235	*N* = 1,158
Median Age (Range)	63 (21–80)	62.6 (25.4–86.1)	60.5 (52.0–67.9)
Sex (M/F)	81/36	135/100	652/506
MGMT Status	65.8% Methylated	NA	40.6% Methylated
Preoperative Karnofsky Performance Status (Mean/SD or Median/Range)	91.61 ± 7.29	NA	90 (80–90)
Preoperative Tumor Volume	Total tumor volume (CE + NCE): Class 1 (Median volumes (Range)): 40.5 cm^3^ (6.6-399.4) Class 2a: 64.3 cm^3^ (20.3-148.5) Class 2b: 51.9 cm^3^ (12.0-340.2)	Tumor volume (CE) Class 2a (Median volumes (IQR): 17.2 cm^3^ (6.7–34.2) Class 2b: 25.9 (10.5–57.9) Class 3a: 36.4 (13.7–52.5) Class 3b: 65.9 (50.8–95.4) Class 4: 17.0 (10.0-37.4)	NA
IDH1 Status	IDH-wildtype only	Patients with IDH1 mutation excluded	IDH-wildtype only
Adjuvant Treatment	Stupp protocol for all patients	53.6% Stupp protocol	Stupp protocol as standard
RT + Concomitant TMZ	All patients	56.2% full regimen	All patients
Chemotherapy Alone	5 patients unable to tolerate chemo	23.4% received either concomitant or adjuvant	NA

### Overall survival stratified by RANO classes

**Fig. 2:**
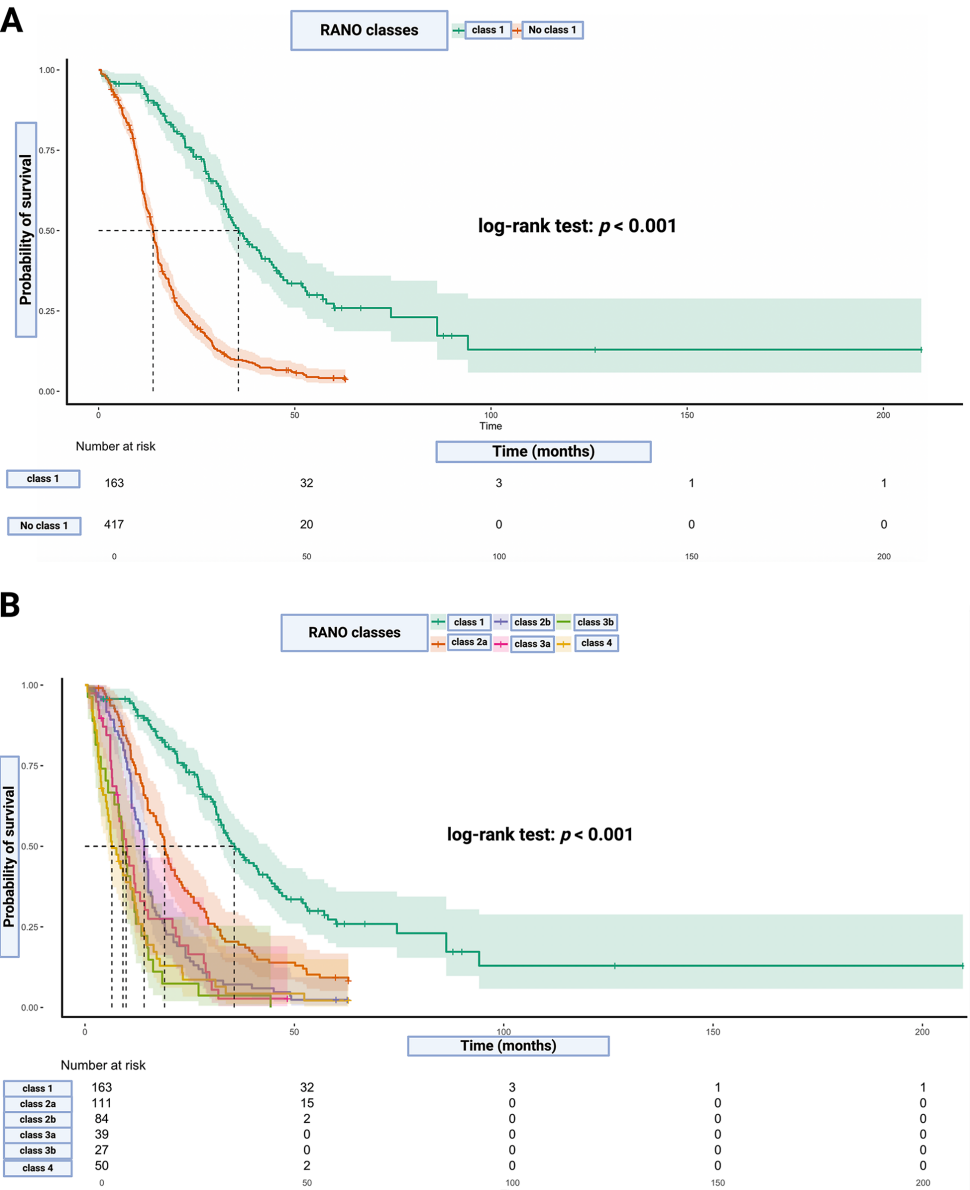
Kaplan-Meier Survival Curves Stratified by RANO Classes. **A**: Kaplan-Meier survival curves comparing OS between patients in Class 1 (supramaximal resection) and No Class 1 (other resection classes combined). The log-rank test shows a significant survival advantage for Class 1 (*p* < 0.001). Shaded areas represent 95% confidence intervals, and the number of patients at risk is displayed below the plot. **B**: Kaplan-Meier survival curves for OS across six RANO classes: Class 1, Class 2a, Class 2b, Class 3a, Class 3b, and Class 4. The survival probabilities differ significantly between these groups (log-rank test *p* < 0.001), demonstrating the prognostic value of detailed stratification. Confidence intervals are shaded for each class, and the number of patients at risk over time is listed below the plot. Both panels highlight the critical impact of EOR on survival, with supramaximal resection (Class 1) showing the longest survival

IPD reconstruction of OS data was possible in 580 patients. Median (Interquartile range) follow-up time of the total cohort was 15.6 (10.1–28.8) months. One-hundred-sixty-three patients underwent a supramaximal RANO class 1 resection with a median survival time of 35.6 months (95% CI: 30.9–40.4). The 12-, 18-, and 24-month OS probabilities in RANO class 1 resected GB patients were 92.4%, 83.7%, and 73.0%. The comparison with all other 417 patients who underwent no RANO class 1 (Classes 2a, 2b, 3a, 3b, 4) resection revealed a significant longer OS probability for those who underwent RANO class 1 resections (log-rank test: *p* < 0.001). The median survival time of those patients classified as RANO classes 2 (a, b), 3 (a, b) or 4 was 13.9 months (95% CI: 13.0-14.7). The 12-, 18-, and 24-month OS probabilities in patients not classified as RANO class 1 resected were 58.5%, 33.3%, and 21.3%. Figure 2a demonstrates the Kaplan-Meier plots illustrating these data. Further comparisons were made between the subgroups within each RANO class (2a, 2b, 3a, 3b, 4). One-hundred-and-eleven patients underwent a RANO class 2a resection with a median survival time of 19.0 months (95% CI: 16.9–21.1). The 12-, 18-, and 24-month OS probabilities in RANO class 2a resected GB patients were 74.2%, 54.7%, and 36.2%. Eighty-four patients underwent a RANO class 2b resection and the median OS time was 14.1 months (95% CI: 12.3–15.9). The OS probabilities at 12, 18, and 24 months for glioblastoma patients who underwent RANO class 2b resection were 59.5%, 27.4%, and 17.9%, respectively. RANO class 3a and 3b resected GB patients had median survival times of 9.8 months (95% CI: 7.1–12.5) and 9.1 months (95% CI: 7.2–10.9), respectively. The overall survival (OS) probabilities at 12, 18, and 24 months were 35.7%, 27.5%, and 19.2%, respectively, for patients who underwent RANO class 3a resections, compared to 29.6%, 11.1%, and 7.4% for those with RANO class 3b resections. Figure 2b demonstrates the OS probabilities of all RANO classes.

### Subgroup analysis of IDH1 wild-type glioblastomas

Further validation of the results was performed with exclusion of the study by Bjorland et al. [12] to ensure that the findings are stringent even in a cohort, which includes only patients who were all routinely tested for IDH1 mutations. The number of patients of RANO class 1 resected group is not altered in this subgroup analysis because the study by Bjorland et al. [12] provided no RANO class 1 data. This subgroup analysis of proven IDH1 wild-type GBs included 345 patients. RANO class 2a resection patients had median survival time of 17.0 months (95% CI: 13.8–20.1) and OS probabilities at 12-, 18-, and 24-month were 71.0%, 41.9%, and 25.8%, respectively. Log-rank test comparing the median OS of RANO class 1 resected IDH1 wild-type GB patients (35.6 months (95% CI: 30.9–40.4)) showed a significant longer OS of these patients compared to RANO class 2a resected GB patients (*p* < 0.001), too. The median OS probabilities of RANO class 2b resected GB patients was 15.1 months (95% CI: 13.6–16.5). Supplementary Fig. 1 summarizes the results.

### Subgroup analysis of each RANO classes

**Fig. 3:**
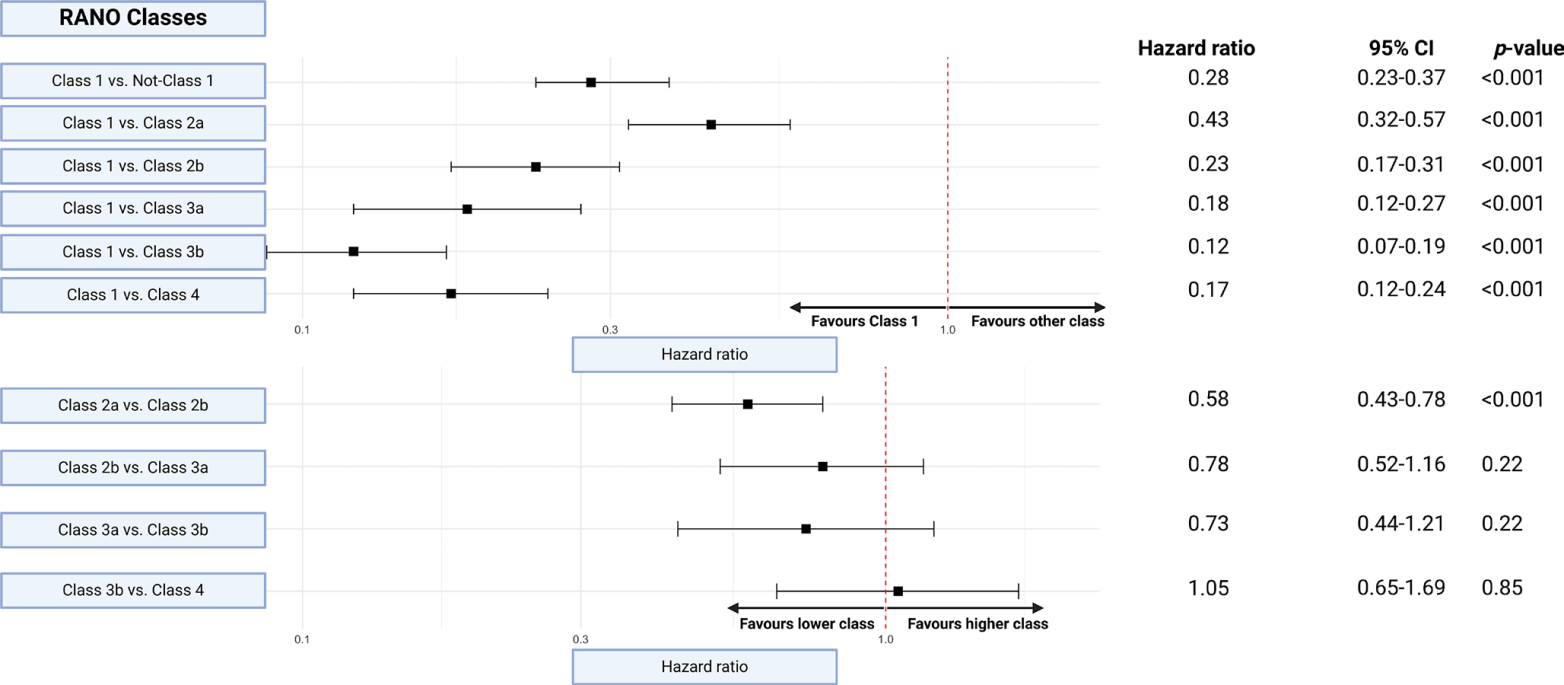
Forest Plot of Hazard Ratios Comparing RANO Classes. This figure presents hazard ratios (HRs) with 95% confidence intervals (CIs) for survival outcomes, comparing various RANO resection classes. In the top panel, comparisons are made between Class 1 (supramaximal resection) and other RANO classes, including aggregated Non-Class 1 (HR: 0.28, 95% CI: 0.23–0.37). Hazard ratios consistently favor Class 1, indicating significantly improved survival compared to other classes. Notably, Class 1 shows a stronger survival benefit over Class 3b (HR: 0.12, 95% CI: 0.07–0.19) and Class 4 (HR: 0.17, 95% CI: 0.12–0.24). In the bottom panel, comparisons between intermediate RANO classes are shown. Noteworthy findings include Class 2a outperforming Class 2b (HR: 0.58, 95% CI: 0.43–0.78), suggesting a survival benefit for complete over near-total resection. Differences between other pairs, such as Class 3a vs. Class 3b, do not reach statistical significance. The dashed red vertical line at HR = 1 marks no effect, where hazard ratios to the left favor the first class in each comparison

Univariable Cox regression analysis revealed significantly reduced hazards of death for RANO class 1 compared to all other RANO classes. Specifically, RANO class 1 had HRs of 0.43 (95% CI: 0.32–0.57) when compared to class 2a, and similarly favorable comparisons against RANO classes 2b, 3a, 3b, and 4 (*p* < 0.001 for all comparisons). The strongest survival advantage was observed when comparing class 1 to class 4 (HR: 0.12, 95% CI: 0.07–0.19). Further comparisons among non- RANO class 1 categories were performed and it was found that RANO class 2a was associated with better survival outcomes than class 2b (HR: 0.58, 95% CI: 0.43–0.78, *p* < 0.001). No significant differences were found between class 2b and 3a, 3a and 3b, or 3b and 4, as their HRs crossed the null line. These results underscore the survival benefits of achieving a supramaximal (class 1) resection. Figure 3 displays Forest plots showing each comparison with RANO class 1 and also the consecutive comparison of RANO resection classes with their neighboring RANO classes.

### Bias and quality evaluation

**Fig. 4:**
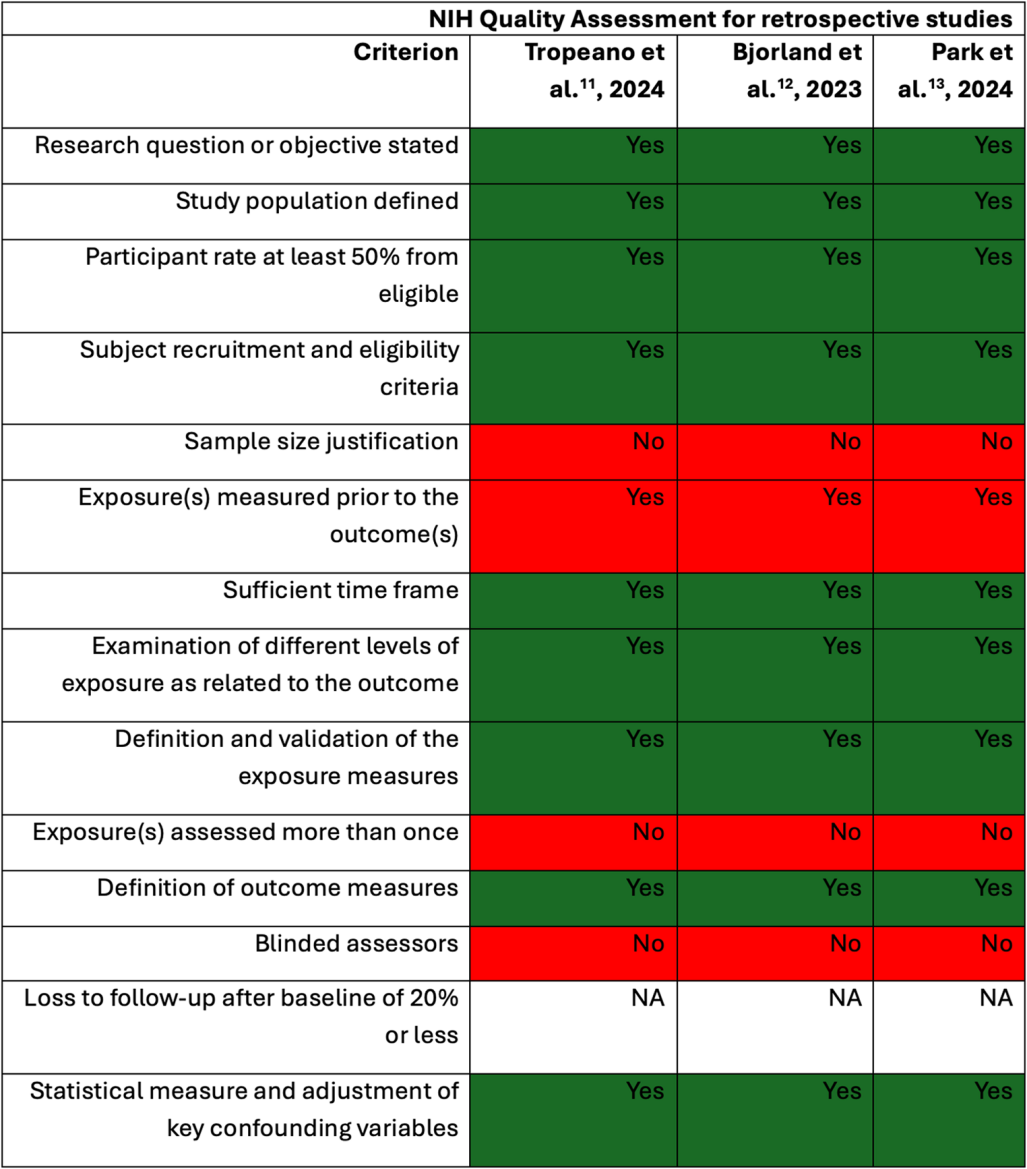
NIH Quality Assessment of Retrospective Studies. This figure displays the quality assessment of various retrospective studies based on the NIH criteria. Each study is evaluated across several methodological parameters, such as whether the research question was clearly stated, if the study population was well defined, and if sufficient follow-up and confounding adjustments were made. Key categories include: Each study is marked with a “Yes” or “No” based on whether the study meets the criteria. “NA” (Not Applicable) is used when certain criteria do not apply to specific studies

The quality assessment of three retrospective studies—Tropeano et al. [11], 2024, Bjorland et al. [12], 2023, and Park et al. [13], 2024—highlights strengths and limitations in their methodological rigor. All studies clearly stated research objectives, defined their study populations, and achieved participant rates above 50% of eligible cases, ensuring adequate sample representation. They also measured exposures prior to outcomes and provided sufficient follow-up periods to assess survival, a critical outcome in glioblastoma research. However, none of the studies justified their sample sizes, which may affect the robustness of statistical power. Additionally, exposure measurements were well-defined, but they were not reassessed over time, potentially limiting the accuracy of longitudinal data. None of the studies employed blinded outcome assessors, introducing a risk of bias in the results. Despite these limitations, all studies adjusted for key confounders in their statistical analyses, enhancing the reliability of their conclusions regarding the prognostic impact of EOR. Further details are summarized in Fig. 4.

## Discussion

The present study confirms and validates the prognostic relevance of the RANO classification for glioblastoma resection, with supramaximal resection (class 1) conferring a significant survival advantage compared to less extensive resections. The present data from 580 patients showed that RANO class 1 resected GB patients had a median OS of 35.6 months compared to 17.0 months in those who underwent RANO class 2a resections. Our findings align with and revalidate those of Karschnia et al. [5], who similarly demonstrated improved median OS for supramaximal resection (RANO class 1) compared with maximal (RANO class 2a resections (Median OS times: 29 vs. 16 months)​. Likewise, Bjorland et al. [12] observed enhanced survival following complete contrast-enhancing resection (class 2a) compared to near-total resection (class 2b), with median OS of 20 and 11 months, respectively​. This concordance across diverse cohorts emphasizes the utility of volumetric classifications in stratifying glioblastoma outcomes and revalidates the robustness of the findings from Karschnia et al. [5]. Hence, the RANO resection categories for EoR should be the benchmark for future studies to enhance comparisons of survival data among the published literature.

Comparing with other recent conventional (analysis of dichotomous data and pooling of effect measures) meta-analyses of supramaximal resection techniques, the present findings further support the survival benefit of resection beyond gross total removal [14–16]. Specifically, Baik et al. [14] highlighted a median OS improvement of 10 months with supramaximal resections without increased neurological morbidity, a critical consideration in surgical planning​.

Despite the consistency in survival benefits, methodological differences must be acknowledged. For instance, some studies employed semi-automated volumetric assessments, while others relied on manual or visual EoR assessment, introducing potential bias [11–13]. Additionally, Roder et al. [17] critiqued the reliance on residual volume thresholds without robust functional assessments, advocating for multimodal approaches incorporating intraoperative MRI​.

A limitation of our study, as with prior analyses, is its retrospective nature, subjecting results to selection bias. Furthermore, variations in adjuvant therapy and genetic profiles, such as MGMT methylation status, complicate direct comparisons. Notably, methylated MGMT tumors consistently exhibit longer OS, underscoring the need for stratified analyses​ [17]. A recent study of 345 patients showed that DNA methylation was linked to better survival, particularly in the receptor tyrosine kinase I and II subclasses [18]. Furthermore, Roder et al. [17]. found in a multicenter prospective study comparing intraoperative MRI with 5-ALA that residual CE tumor portions (0 cm [3] vs. > 0 cm [3]) had no influence on the OS in MGMT methylated GBs.

Prospectively, the ongoing SUPRAMAX trial offers an opportunity to validate these findings within a rigorously controlled framework in a multicentre prospective two-arm observational cohort study. This multicenter study aims to compare supramaximal and maximal resection techniques concerning OS and neurological functioning until 6 months postoperatively [19]. Such data will be invaluable in refining surgical guidelines and assessing the risk-benefit balance of extensive supramaximal resections in glioblastoma.

Future research should also explore integrating advanced imaging modalities, including FLAIR and PET, to better delineate tumor margins and infiltrative zones. Positron emission tomography is not widely available but recent studies have also suggested that its integration in surgical planning and the supramaximal resection including areas with [11]C-met uptake beyond the CE tumor enhances survival in GB [20]. Furthermore, the development of deep-learning based algorithms for fully automated volumetric analysis could enhance standardization across centers, as variability in resection extent definitions remains a significant barrier to broad applicability​ [21].

### Limitations & prospect

Despite this meta-analysis demonstrates data from 580 patients revalidating the robustness of the RANO resect classification, this individual patient data meta-analysis has several limitations. First, the included data cannot be stratified according to the MGMT promotor methylation status. Second, also clinical important variables such as age and KPS cannot be stratified among the reconstructed IPD [22, 23]. However, the studies of Tropeano et al. [11] and Park et al. [13] included IDH-wild-type GBs only to be in line with the current WHO grading, and these two studies provided the IPD for RANO class 1 resected patients [24]. The study by Bjorland et al. [12] provided IPD from RANO resection classes 2a-4 and did not routinely check for IDH mutational status, but they conducted it in younger patients and those with histological features of low-grade glioma. Therefore, the likelihood of potentially including IDH-mutant gliomas among RANO classes 2–4 is very low and no potential IDH-mutant patient are in the RANO class 1 (supramaximal resection) resected patient groups from the studies by Tropeano et al. [11] and Park et al. [13]. However, the RANO class 1 resected patients of the study by Park et al. [13] are extracted from their grade 1 resection system, which implicates that these patients have MGMT promotor methylated tumors and are ≤ 65 years at diagnosis. Furthermore, subgroup analysis of patients who all underwent diagnostic screening for IDH1 mutations reconfirmed the total cohort (see supplementary Fig. 1). The present study revalidates the use of the RANO resect classes and might introduce the design of new future important studies on the role of intraoperative imaging in high-grade glioma surgery because of the limited role of current benchmark intraoperative imaging method 5-ALA for NCE FLAIR abnormal tumor portions without anaplastic foci [25, 26]. Furthermore, future resection classification systems prognosticating OS outcome might also benefit from the inclusion of molecular and demographic characteristics.

## Conclusion

In conclusion, our study reaffirms the prognostic utility of the RANO classification and highlights the survival advantage conferred by supramaximal resections. However, the heterogeneity of existing evidence and the absence of randomized controlled trials underscore the necessity for continued investigation in this domain. The integration of functional preservation metrics and advanced imaging will likely be pivotal in optimizing glioblastoma management.

## Electronic supplementary material

Below is the link to the electronic supplementary material.

**Supplementary Material 1:** Supplementary Figure 1

**Supplementary Material 2:** Supplementary methods 1

**Supplementary Material 3:** Supplementary methods 2

**Supplementary Material 4:** Supplementary methods 3

## Data Availability

No datasets were generated or analysed during the current study.
