# Supplementary material for: Prognostic revalidation of RANO categories for extent of resection in glioblastoma: a reconstruction of individual patient data
Source: J Neurooncol. 2025 Feb 24;172(3):515–25. doi: 10.1007/s11060-025-04950-0 (PMC11968501; doi:10.1007/s11060-025-04950-0)
Supplement: Supplementary file 2 — Supplementary Material 2: Supplementary methods 1 [file 11060_2025_4950_MOESM2_ESM.docx]

| **Supplementary table 1:** PRISMA Checklist for Systematic Review and Meta-Analysis on Revalidation of RANO resect classes for glioblastoma | | | |
| --- | --- | --- | --- |
| **Section** | **Item** | **Description** | **Page Number** |
| **Title** | Title | Prognostic Revalidation of RANO Categories for Extent of Resection in Glioblastoma: A Reconstruction of Individual Patient Data | 1 |
| **Abstract** | Structured Summary | Abstract includes background, objectives, data sources, study eligibility criteria, methods, results, and conclusions | 3 |
| **Introduction** | Rationale | Importance of EOR and revalidation of RANO resection categories to improve survival in glioblastoma | 4 |
| **Introduction** | Objectives | To revalidate RANO resection classes and analyze survival outcomes based on reconstructed IPD | 4 |
| **Methods** | Protocol and Registration | Adhered to PRISMA guidelines; registered in PROSPERO | 5-6 |
| **Methods** | Eligibility Criteria | Inclusion criteria: adult glioblastoma patients with detailed OS and RANO-based EOR data | 5-6 |
| **Methods** | Information Sources | PubMed, Google Scholar, and Cochrane Library searched between August 2022–November 2024 | 5-6 |
| **Methods** | Search Strategy | Keywords: “glioblastoma,” “RANO resect,” “extent of resection,” “survival” | 5-6 |
| **Methods** | Selection Process | Two reviewers independently screened studies; discrepancies resolved by a third reviewer | 5-6 |
| **Methods** | Data Collection Process | IPD reconstructed using Digitizelt and IPDfromKM; extracted data on OS, EOR, and patient characteristics | 5-6 |
| **Methods** | Data Items | Variables: age, sex, MGMT methylation, KPS, EOR, adjuvant radiotherapy | 5-6 |
| **Methods** | Risk of Bias Assessment | NIH-QAT applied to assess study quality | 5-6 |
| **Methods** | Effect Measures | Kaplan-Meier survival curves and Cox regression to determine hazard ratios | 5-6 |
| **Methods** | Synthesis of Results | Subgroup analyses for survival based on RANO resect classes | 5-6 |
| **Results** | Study Selection | Flowchart showing study selection (339 identified, 3 included) | 7 |
| **Results** | Study Characteristics | Summary of key characteristics of included studies | 8-9 |
| **Results** | Results of Individual Studies | OS stratified by RANO classes in each study | 8-9 |
| **Results** | Synthesis of Results | Pooled OS data; Kaplan-Meier survival estimates for RANO classes | 9-13 |
| **Results** | Risk of Bias in Studies | Quality assessment using NIH-QAT summarized | 13-14 |
| **Discussion** | Summary of Evidence | Revalidation of RANO classifications and their prognostic impact | 16-17 |
| **Discussion** | Limitations | Retrospective design, lack of molecular stratification, and variability in patient characteristics | 16-17 |
| **Discussion** | Conclusions | RANO classifications validated as effective prognostic tools; future trials like SUPRAMAX are needed | 17 |
| **Funding** | Funding | No external funding reported | 18 |
| **Acknowledgments** | Contributions | Authorship and use of BioRender for visuals acknowledged | 18 |
