# Supplementary material for: Prognostic revalidation of RANO categories for extent of resection in glioblastoma: a reconstruction of individual patient data
Source: J Neurooncol. 2025 Feb 24;172(3):515–25. doi: 10.1007/s11060-025-04950-0 (PMC11968501; doi:10.1007/s11060-025-04950-0)
Supplement: Supplementary file 3 — Supplementary Material 3: Supplementary methods 2 [file 11060_2025_4950_MOESM3_ESM.docx]

**Literature Search Summary for Systematic Review on Glioblastoma Surgical Outcomes**

This document outlines the comprehensive literature search methodology used for a systematic review on glioblastoma surgical outcomes, focusing on the role of the RANO classification, extent of resection, and related variables. Searches were conducted across PubMed, Google Scholar, and the Cochrane Library to identify relevant studies published after August 12, 2022. The search was limited to November 1, 2024.

**1. PubMed Search**

**Search Syntax:**

javascript

Code kopieren

(("RANO classification"[Title/Abstract] OR "RANO categories"[Title/Abstract] OR "extent of resection"[Title/Abstract])

AND ("glioblastoma"[MeSH Terms] OR "glioblastoma"[Title/Abstract])

AND ("surgery"[MeSH Terms] OR "resection"[Title/Abstract]))

AND ("2022/08/12"[Date - Publication] : "3000"[Date - Publication])

**Search Focus:**

- Identification of studies discussing RANO classification or extent of resection in glioblastoma.
- Studies were filtered by publication date to ensure the latest evidence was included.

**2. Google Scholar Search**

**Search Syntax:**

arduino

Code kopieren

"RANO classification" OR "RANO categories" OR "extent of resection" AND "glioblastoma" AND "surgery" OR "resection" after:2022-08-12

**Search Focus:**

- Relevant studies involving glioblastoma surgical outcomes and the RANO classification.
- Due to practical limitations, only the first 200 results were manually screened for inclusion.

**3. Cochrane Library Search**

**Search Syntax:**

sql

Code kopieren

("RANO classification" OR "RANO categories" OR "extent of resection")

AND ("glioblastoma")

AND ("surgery" OR "resection")

WITH Publication Year from 2022 to 2024

**Search Focus:**

- Studies assessing surgical resection strategies in glioblastoma, categorized under RANO or extent of resection.
- Search refined to publications within the specified date range to ensure contemporary relevance.

**Search Rationale:**
These search strategies were designed to capture a broad yet focused dataset on glioblastoma surgical management, particularly under RANO classification. The specified time frame ensures only the most recent and applicable studies are included in the review.
