# Supplementary material for: Prognostic revalidation of RANO categories for extent of resection in glioblastoma: a reconstruction of individual patient data
Source: J Neurooncol. 2025 Feb 24;172(3):515–25. doi: 10.1007/s11060-025-04950-0 (PMC11968501; doi:10.1007/s11060-025-04950-0)
Supplement: Supplementary file 4 — Supplementary Material 4: Supplementary methods 3 [file 11060_2025_4950_MOESM4_ESM.docx]

**Study Protocol for Systematic Review and Meta-Analysis on RANO Classification and Extent of Resection in Glioblastoma**

**Title**
Application and Prognostic Value of RANO Classification in Glioblastoma Surgery: A Systematic Review and Individual Patient Data Meta-Analysis

**Background**

Glioblastoma is the most aggressive primary brain tumor, with a median survival of only 15 months despite aggressive multimodal treatment​ (1, 2). Surgical resection plays a pivotal role in its management, as maximizing the extent of resection (EoR) has been shown to significantly improve both overall survival (OS) and progression-free survival (PFS)​(3). However, inconsistent terminology and varying criteria for EoR across studies have historically limited comparative analyses and clinical decision-making​ (2).

The recent RANO (Response Assessment in Neuro-Oncology) classification system introduces standardized categories for assessing EoR, encompassing both contrast-enhancing (CE) and non-contrast-enhancing (non-CE) tumor volumes. Studies have demonstrated that removing non-CE tumor tissue beyond the traditional CE margins—so-called supramaximal or supramarginal resections—may further enhance survival outcomes ​(1). For example, supramaximal resection has been associated with a significant increase in PFS without an increased risk of perioperative complications​ (1). However, prospective randomized data is not available yet and is subject of ongoing randomized trials (4)

Moreover, the prognostic utility of residual tumor volume, particularly residual non-CE tumor, has been validated in several studies, highlighting the importance of a comprehensive resection strategy​ (2). However, the precise thresholds for residual tumor volumes and their survival impact remain a topic of ongoing research. This systematic review and meta-analysis aim to synthesize the available evidence on RANO classification and its prognostic value in glioblastoma, providing clarity on optimal surgical strategies and their impact on survival ​(1).

**Objectives**

1. To evaluate OS and PFS based on RANO classes in glioblastoma patients.
2. To assess the prognostic utility of RANO classification in predicting survival outcomes.
3. To conduct an IPD meta-analysis for more precise survival estimates.

**Methods**

**1. Study Design**
This systematic review and meta-analysis will be conducted in accordance with PRISMA guidelines (5). Individual patient data (IPD) will be reconstructed from Kaplan-Meier curves with number at risk tables using DigitezIt software (Version 2.5.10 for macOS) and using the R package IPDfromKM (6, 7).

**2. Inclusion/Exclusion Criteria**

**Inclusion Criteria:**

- Studies applying the RANO classification or providing volumetric resection data for glioblastoma (Both: T1 contrast-weighted and FLAIR MR-images)
- Reporting survival outcomes (OS).
- Published in English from August 12, 2022 - November 1, 2024.

**Exclusion Criteria:**

- Studies focusing on non-glioblastoma tumors.
- Reviews, editorials, conference abstracts without original data.

**3. Study Selection**

- Titles and abstracts will be screened independently by two reviewers.
- Full-text articles will be reviewed for eligibility.
- Disagreements will be resolved by consensus or consultation with a third reviewer.

**4. Data Extraction**
A standardized data extraction form will be used to collect:

- Study characteristics (author, year, country, design).
- Patient demographics (age, sex, tumor characteristics).
- RANO classification application.
- Outcomes: OS, PFS, and other survival metrics.

**IPD Reconstruction:**
IPD will be reconstructed from Kaplan-Meier curves using the IPDfromKM R package​(7).

**Risk of Bias Assessment**

The **NIH Quality Assessment Tool** will be used to evaluate methodological rigor and potential bias in observational studies​(8).

**Data Synthesis**

A synthesis of the reconstructed IPD will be conducted, and the data will be analyzed with log-rank tests and univariable Cox proportional hazard models.

- Pooled hazard ratios for OS and PFS.
- Kaplan-Meier curves stratified by RANO classes.

**References**

1. Wach J, Vychopen M, Kühnapfel A, Seidel C, Güresir E. A Systematic Review and Meta-Analysis of Supramarginal Resection versus Gross Total Resection in Glioblastoma: Can We Enhance Progression-Free Survival Time and Preserve Postoperative Safety? Cancers (Basel). 2023 Mar 15;15(6):1772. doi: 10.3390/cancers15061772.Wach et al., Cancers 2023​(cancers-15-01772 (1)).
2. Karschnia P, Young JS, Dono A, Häni L, Sciortino T, Bruno F, Juenger ST, Teske N, Morshed RA, Haddad AF, Zhang Y, Stoecklein S, Weller M, Vogelbaum MA, Beck J, Tandon N, Hervey-Jumper S, Molinaro AM, Rudà R, Bello L, Schnell O, Esquenazi Y, Ruge MI, Grau SJ, Berger MS, Chang SM, van den Bent M, Tonn JC. Prognostic validation of a new classification system for extent of resection in glioblastoma: A report of the RANO resect group. Neuro Oncol. 2023 May 4;25(5):940-954. doi: 10.1093/neuonc/noac193. Liu et al., BMC Med Res Methodol 2021​(s12874-021-01308-8).
3. Roder C, Stummer W, Coburger J, Scherer M, Haas P, von der Brelie C, Kamp MA, Löhr M, Hamisch CA, Skardelly M, Scholz T, Schipmann S, Rathert J, Brand CM, Pala A, Ernemann U, Stockhammer F, Gerlach R, Kremer P, Goldbrunner R, Ernestus RI, Sabel M, Rohde V, Tabatabai G, Martus P, Bisdas S, Ganslandt O, Unterberg A, Wirtz CR, Tatagiba M. Intraoperative MRI-Guided Resection Is Not Superior to 5-Aminolevulinic Acid Guidance in Newly Diagnosed Glioblastoma: A Prospective Controlled Multicenter Clinical Trial. J Clin Oncol. 2023 Dec 20;41(36):5512-5523. doi: 10.1200/JCO.22.01862.
4. Gerritsen JKW, Young JS, Chang SM, Krieg SM, Jungk C, van den Bent MJ, Satoer DD, Ille S, Schucht P, Nahed BV, Broekman MLD, Berger M, De Vleeschouwer S, Vincent AJPE. SUPRAMAX-study: supramaximal resection versus maximal resection for glioblastoma patients: study protocol for an international multicentre prospective cohort study (ENCRAM 2201). BMJ Open. 2024 Apr 29;14(4):e082274. doi: 10.1136/bmjopen-2023-082274
5. Stewart LA, Clarke M, Rovers M, Riley RD, Simmonds M, Stewart G, Tierney JF; PRISMA-IPD Development Group. Preferred Reporting Items for Systematic Review and Meta-Analyses of individual participant data: the PRISMA-IPD Statement. JAMA. 2015 Apr 28;313(16):1657-65. doi: 10.1001/jama.2015.3656.
6. Rakap S, Rakap S, Evran D, Cig O. Comparative evaluation of the reliability and validity of three data extraction programs: UnGraph, GraphClick, and DigitizeIt. Comput Hum Behav. 2016;55:159-66. doi: 10.1016/j.chb.2015.09.008.
7. Liu N, Zhou Y, Lee JJ. IPDfromKM: reconstruct individual patient data from published Kaplan-Meier survival curves. BMC Med Res Methodol. 2021 Jun 1;21(1):111.
8. Ma LL, Wang YY, Yang ZH, Huang D, Weng H, Zeng XT. Methodological quality (risk of bias) assessment tools for primary and secondary medical studies: what are they and which is better? Mil Med Res. 2020 Feb 29;7(1):7. doi: 10.1186/s40779-020-00238-8.


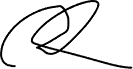


Priv.-Doz. Dr. med. Johannes Wach, MBA

Department of Neurosurgery

University Hospital Leipzig

Leipzig University

Liebigstraße 20, 04103 Leipzig, Germany
